# Application of Iron Oxide Nanoparticles in the Diagnosis and Treatment of Neurodegenerative Diseases With Emphasis on Alzheimer’s Disease

**DOI:** 10.3389/fncel.2020.00021

**Published:** 2020-02-28

**Authors:** Shen Luo, Chi Ma, Ming-Qin Zhu, Wei-Na Ju, Yu Yang, Xu Wang

**Affiliations:** ^1^Department of Neurology and Neuroscience Center, The First Hospital of Jilin University, Changchun, China; ^2^Department of Critical Care Medicine, The First Affiliated Hospital, Sun Yat-sen University, Guangzhou, China; ^3^Department of Neurosurgery, The First Hospital of Jilin University, Changchun, China

**Keywords:** iron oxide nanoparticle, magnetic resonance imaging, Alzheimer’s disease, amyloid-β, neurodegeneration

## Abstract

Neurodegenerative diseases are characterized by chronic progressive degeneration of the structure and function of the nervous system, which brings an enormous burden on patients, their families, and society. It is difficult to make early diagnosis, resulting from the insidious onset and progressive development of neurodegenerative diseases. The drugs on the market cannot cross the blood–brain barrier (BBB) effectively, which leads to unfavorable prognosis and less effective treatments. Therefore, there is an urgent demand to develop a novel detection method and therapeutic strategies. Recently, nanomedicine has aroused considerable attention for diagnosis and therapy of central nervous system (CNS) diseases. Nanoparticles integrate targeting, imaging, and therapy in one system and facilitate the entry of drug molecules across the blood–brain barrier, offering new hope to patients. In this review, we summarize the application of iron oxide nanoparticles (IONPs) in the diagnosis and treatment of neurodegenerative disease, including Alzheimer’s disease (AD), Parkinson’s disease (PD), and amyotrophic lateral sclerosis (ALS). We focus on IONPs as magnetic resonance imaging (MRI) contrast agents (CAs) and drug carriers in AD. What most neurodegenerative diseases have in common is that hall marker lesions are represented by protein aggregates (Soto and Pritzkow, 2018). These diseases are of unknown etiology and unfavorable prognosis, and the treatments toward them are less effective (Soto and Pritzkow, 2018). Such diseases usually develop in aged people, and early clinical manifestations are atypical, resulting in difficulty in early diagnosis. Recently, nanomedicine has aroused considerable attention for therapy and diagnosis of CNS diseases because it integrates targeting, imaging, and therapy in one system (Gupta et al., 2019). In this review article, we first introduce the neurodegenerative diseases and commonly used MRI CAs. Then we review the application of IONPs in the diagnosis and treatment of neurodegenerative diseases with the purpose of assisting early theranostics (therapy and diagnosis).

## Introduction

### Introduction of Neurodegenerative Diseases

#### Introduction of Alzheimer Disease (AD)

The main characteristic of Alzheimer’s disease (AD) is the progressive deterioration of cognitive function, most commonly the loss of memory, increasingly influencing patients’ activity of daily living and leading to loss of independency (Tiwari et al., 2019). The hallmark histological abnormalities of AD comprise of the extracellular aggregation of amyloid plaques and fibrillar aggregates of the microtubule associated with tau protein (Tiwari et al., 2019). The deposition of amyloid plaque, which is caused by the mounting production, accumulation, and aggregation of the amyloid-β (Aβ), is the primary histopathological characteristic of AD (Tiwari et al., 2019). The most widely accepted AD etiology is the amyloid cascade hypothesis (Barage and Sonawane, 2015). The incorrect process of the Aβ protein precursor (AβPP) by γ-secretase, which gives rise to the pathological 40–42 amino acid long cleaved peptide, known as Aβ, is fundamental to this hypothesis (Chen and Mobley, 2019; Tiwari et al., 2019). The excess of Aβ finally results in the aggregated fibrils of plaques and neurotoxic oligomers (Chen and Mobley, 2019).

Currently for AD, the early diagnosis could provide treatment opportunities to high-risk groups, and the way to cure diseases under development seems to be proven effective only at the very early stages of the initiated amyloid deposition (Frisoni et al., 2017). For patients in the earlier stage of AD, the correct diagnosis and treatment would enable to delay cognitive impairment and irreversible neuronal damage (Frisoni et al., 2017). Therefore, there is a rising demand to develop reliable early detective tools for AD.

#### Introduction of Parkinson’s Disease (PD) and Other Neurodegenerative Diseases

Parkinson’s disease (PD) is the second most common neurodegenerative disease in the whole world (Balestrino and Schapira, 2019). PD mainly affects the locomotor system by degenerating the neuron, finally leading to severe disability (Balestrino and Schapira, 2019). The clinical criteria for diagnosis of PD depend mainly on the observation of movement disorders such as cogwheel rigidity, bradykinesia, resting tremor, etc. (Balestrino and Schapira, 2019). In the dopaminergic neurons, phosphor-α-synuclein molecules can aggregate with one another easily to form Lewy body, and these degenerative dopaminergic neurons lose the function of expressing dopamine, finally leading to the damage of the motor cortex and movement disorders (Ikenaka et al., 2019). Thus, for PD, α-synuclein is the most acknowledged biomarker (Ikenaka et al., 2019). If the quantity of degenerative dopaminergic neurons in the basal ganglia is over 50%, the clinical features of PD can gradually present. The clinical symptoms of PD might overlap with other movement disorders such as progressive supranuclear palsy (PSP) and multiple system atrophy (MSA; Giagkou and Stamelou, 2018). Therefore, going by clinical manifestations only, early PD diagnosis is difficult and prone to misdiagnosis.

Another neurodegenerative disease, amyotrophic lateral sclerosis (ALS), is an adult-onset neurodegenerative disorder. The major pathological manifestations of ALS are progressive loss of upper motor neurons of the corticospinal tract, lower motor neurons of ventral roots of the spinal cord, and brainstem nuclei (Gagliardi et al., 2019).

For the moment, despite the fact that many kinds of treatment appeared, no treatment has been shown to be effective for neurodegenerative diseases. Mesenchymal stem cell (MSC) therapy is regarded as one of the most prospective approaches for the treatment of neurodegenerative diseases, including PD and ALS (Baloh et al., 2018; Staff et al., 2019). In addition to MSC, neural stem cells (NSCs) could turn to be an optimal selection to replace specific lost neurons *in vitro* and *in vivo* (Sugaya and Vaidya, 2018).

### Common Auxiliary Examination Methods for Neurodegenerative Diseases

#### Autopsy

Currently for AD, there is no other confirmed diagnostic method except autopsy (Pillai et al., 2018). Although researchers use plenty of neuropsychological tests to make the clinical diagnosis, the diagnosis process could be affected by many other factors. So the definitive diagnosis still depends on autopsy with histological and pathological findings of sufficient numbers of plaques (Tiwari et al., 2019). But this method is an invasive test and is not usually accepted by patients.

#### Laboratory Inspection

Cerebrospinal fluid (CSF) is usually collected through the lumbar puncture, which is invasive and uncomfortable. The reduction of CSF Aβ42 and the elevation of CSF tau are the biomarkers of AD. But the elevated CSF tau is just a biomarker of neuronal injury, not specific to AD (Olsson et al., 2016).

Compared to CSF, blood sample is much easier to obtain in clinics (Yang et al., 2016). For PD, α-synuclein can be expressed in the peripheral blood system at very low amounts, but it is not applicable for clinical use due to the poor low-detection limit of blood test (Yang et al., 2016).

Nanotechnology can play an important role in increasing the sensitivity of detection of biomarkers (Gupta et al., 2019). Applications of the magnetic nanoparticles (MNPs) detecting biomarkers for AD and PD are mentioned later in the section The Application of Iron Oxide Nanoparticles (IONPs) in Neurodegenerative Diseases.

#### Positron Emission Tomography (PET)

On positron emission tomography (PET), one of the biomarkers of AD-related synaptic dysfunction is the decreased fluorodeoxyglucose ^18^F (FDG) uptake, which indicates temporoparietal hypometabolism (Chandra et al., 2019). Thus, currently PET is being used to image the amyloid deposition in AD, mild cognitive impairment (MCI), and normal aged controls in human (Chandra et al., 2019). However, in terms of PET, the visualization of individual plaques may be limited by the low spatial resolution for it is not clear enough to detect the earliest stage of amyloid deposition (Wadghiri et al., 2013).

#### Magnetic Resonance Imaging (MRI)

Compared with PET, magnetic resonance imaging (MRI) has a much higher spatial resolution especially for soft tissue and does not require radiotracer injection (Wadghiri et al., 2013). And the instrumentation operating at 1.5–3 T is widely used for patient and animal imaging (Wadghiri et al., 2013). MRI can be used not only for imaging Aβ aggregation in the murine brain but also for monitoring other AD-related morphological and functional alterations (Rotman et al., 2011).

Chamberlain et al. (2009) reported that the susceptibility-weighted imaging (SWI) method can provide high plaque contrast for *ex vivo* plaque imaging, which increases the sharpness of the image in about 1 h 30 min. But *in vivo*, the SWI method has been found impractical due to the magnetic susceptibility artifact from the surface vessels and the air-to-skull interface (Chamberlain et al., 2011).

#### Others

Genetic sequence analysis might be a method for diagnosis of early-stage PD, whereas only about 10% of PD patients are hereditary (Yang et al., 2016).

### Commonly Used MRI Contrast Agents (CAs)

#### Introduction of MRI CAs

The contrast agents (CAs) in MRI can enhance the image contrast of the tissue/regions where they are delivered by shortening the water protons spin−lattice T1 and/or spin−spin T2 relaxation times (Busquets et al., 2014). Therefore, the image is CA affection to the relaxivity of the adjacent water protons through the dipolar interaction predominantly, rather than the CA compound itself (Busquets et al., 2014).

Traditionally, there are two different categories for MRI CAs taking effect in T1 and T2, respectively. T1 CAs can increase the T1 relaxation time, resulting in high signal in T1-weighted images. T2 CAs could reduce the T2 relaxation time, which reduces both T2 and T2* signals and gives rise to dark contrast in T2-weighted images (De et al., 2011). MRI CAs are also divided into “positive” CAs, which are in the majority, and “negative” CAs. In brief, “positive” CAs are the contrasts made by a gadolinium-based compound (paramagnetic CAs) administration, which enhances the MRI intensity of the signal. “Negative” CAs mean iron oxide based on superparamagnetic CAs. Superparamagnetic CAs can commonly decrease the MRI signal of the regions where they are delivered (Busquets et al., 2014).

#### Commonly Used MRI CAs

Currently, the most widely used MRI CAs are gadolinium (Gd^3+^) chelates (Marasini et al., 2020). By using high-field MRI, Gd^3+^ chelates can demonstrate the Aβ plaque imaging both *ex vivo* and *in vivo* (Sillerud et al., 2013). Due to the long electron spin-lattice relaxation time and abundant unpaired electrons (seven) per Gd^3+^ ion has, the relaxation rate of water hydrogen can be increased significantly. However, ionic gadolinium complexes can leak toxic Gd^3+^ ions with short half-life and it has been reported that Gd^3+^ chelates can lead to renal insufficiency (Rashid et al., 2016; Marasini et al., 2020). Fluorinated small molecules that bind to amyloid plaques can be detected by ^19^F MRI (Jirak et al., 2019). But for their low *in vivo* concentrations, this method might be difficult to be applied to human clinical medicine (Sillerud et al., 2013). Besides, gold nanoparticles (Au NPs) present a significant MRI signal change during Aβ self-aggregation, but the intrinsic cytotoxicity caused by Co in preparing the NPs remains to be thoroughly evaluated (Brambilla et al., 2011).

Among all these MRI CAs, pure iron oxides such as magnetite (Fe_3_O_4_) and maghemite (γ-Fe_2_O_3_) are the most common biocompatible magnetic nanomaterials (Ling and Hyeon, 2013). Iron oxides are benign, nontoxic, and tolerated biologically, and they can be injected into the human body and incorporated into human natural processes of metabolism, serving as MRI CAs or drug delivery system (Ling and Hyeon, 2013). Currently, some IONP-based MRI CAs have already been used in clinical trials. Apart from the benefits mentioned above, IONPs offer many important biomedical applications, such as cell tracking, protein separation, and hyperthermia (Ling and Hyeon, 2013).

IONPs usually accumulate in liver and spleen. They can be eliminated by liver, spleen, and kidney (Arami et al., 2015). The potential toxic effects of free iron ions released from IONPs can be blocked by maintaining iron homeostasis. Excess iron ions can combine with ferritin to maintain the iron store, which is involved in many biological processes, including hemoglobin synthesis (Arami et al., 2015). One study has showed that the release of iron ions from IONPs may result in iron accumulation, oxidative stress, and protein aggregation, which are toxic to the neural cell. However, the toxicity levels of IONPs are determined by their properties, including the size, concentration, surface charge, and the type of coating and functional groups (Yarjanli et al., 2017).

Besides, colloidal IONPs such as ultrasmall superparamagnetic iron oxide (USPIO) and superparamagnetic iron oxide (SPIO) have already been used as MRI CAs in targeting drug delivery (Busquets et al., 2014; Dulińska-Litewka et al., 2019). Superparamagnetic iron oxide nanoparticles (SPIONs), with the iron oxide core and magnetic coating, have fundamental features such as high saturation magnetic moment, relatively stable chemical properties, and minimized potential toxicity (Busquets et al., 2014; Dulińska-Litewka et al., 2019). They can improve the MRI sensitivity by serving as *in vivo* or *in vitro* CAs and thus have potential biomedical applications for MRI CAs (Busquets et al., 2014; Dulińska-Litewka et al., 2019). The commonly used MRI CAs are briefly summarized in Table 1.

**Table 1:** Commonly used magnetic resonance imaging (MRI) contrast agents.

	Service conditions	Advantages	Disadvantages
Gadolinium chelate commercial available products: Magnevist^®^ (Gd-DTPA), Dotarem^®^ (Gd-DOTA), Prohance^®^ (Gd-HP-DO3A), Primovist^®^, Eovist^®^ (Gd-EOB-DTPA), etc.	High-field MRI	Increase the relaxation rate of water hydrogen significantly	Leak toxic Gd^3+^ ions; lead to renal insufficiency (Rashid et al., 2016; Marasini et al., 2020)
Fluorinated small molecules	^19^F MRI	The sensitivity of magnetic resonance of ^19^F is relatively high; endogenous ^19^F-derived background noise should be very low; ^19^F can be found abundantly, result in low manufacturing cost.	Low *in vivo* concentrations (Sillerud et al., 2013; Jirak et al., 2019)
Gold nanoparticles (Au NPs)	Regular MRI	Present a significant MRI signals change	Intrinsic cytotoxicity (Brambilla et al., 2011)
Pure iron oxides γ-Fe_2_O_3_ and Fe_3_O_4_	MRI	Benign, nontoxic, tolerated biologically	Naked iron oxide nanocrystals can cause damage to *in vitro* cytotoxicity (Ling and Hyeon, 2013)
Colloidal iron oxide nanoparticles SPIOs USPIOs Commercial available products: Feridex^®^ (Ferumoxides), Resovist^®^ (Ferucarbotran)	MRI	High saturation magnetic moment, relatively stable chemical properties, minimized potential toxicity	The use of organic solvents in the synthetic schemes result in the hydrophobic of SPIONs (Busquets et al., 2014; Dulińska-Litewka et al., 2019)

### The Carrier

#### The Introduction of the Blood–Brain Barrier

The blood–brain barrier (BBB) is mainly formed by neuronal pericytes, perivascular astrocytes, and brain capillary endothelial cells (BCECs). The infrastructure that each BCEC tightly connects with all their neighboring cells can firmly constitute a physical, chemical, and immunological barrier, and thus can keep the central nervous system (CNS) separate from peripheral blood circulation (Teleanu et al., 2019). The BBB permits only a few percent of potential CNS drugs into the CNS (Teleanu et al., 2019). Take MNPs for example; atomic force microscopy demonstrated that when MNPs cross the BBB, they can be internalized by endothelial cells (Kong et al., 2012). The BBB is responsible for the accurate internal regulation of the CNS; thus, any damage of the BBB can be related to systemic inflammatory or immune dysfunction, which further leads to the initiation of several neurodegenerative pathways (Teleanu et al., 2019).

In general, there are two ways of carrying drugs: (1) directly immobilized on the MNPs surface; and (2) tethered *via* polyethylene glycol (PEG) coating or other organic/inorganic polymer layer. The problem is that the free drugs can be eliminated by metabolism (enzymatic mainly) easily before they reach the target (Ding et al., 2014).

There has been a study examining the biodistribution of IONPs using lysophosphatidic acid (LPA) to help the entry to the brain. IONPs without LPA coating were mainly deposited in liver and spleen, with a plasma half-life of 6 min. The accumulation amount of IONPs with LPA coating in the brain and spleen increased approximately 4-fold compared with the control. Mice treated with LPA and IONP showed no sign of peripheral immune cell passing through the BBB and minimal activation of microglia and astrocytes, indicating a safe and effective strategy for IONP delivery to the brain (Sun et al., 2016).

CAs or therapeutic drugs can be delivered from the olfactory mucosa to the brain by neuronal cells through the olfactory pathway, which consists of olfactory epithelium in nasal cavity, lamina propria, and olfactory bulb in the CNS (Khan et al., 2017). Since the nasal passage is the only direct connection between the external environment and the brain, it provides an applicable method for CAs or therapeutic drugs to enter the brain by bypassing the BBB rather than to cross it (Salama et al., 2017). Thus, the olfactory pathway is more efficient for reducing hepatic/renal clearance and the systemic exposure (Akilo et al., 2016; Khan et al., 2017).

#### The Function of the Carrier

For the carriers whose sizes fall within the range from 120 to 200 nm, they can pass the trap of the reticuloendothelial system (RES) easily and cannot be detected by the cells of liver and spleen. This can prolong the time that the carriers remain in contact with the BBB, thus increasing the odds for the drug to be absorbed by the CNS ultimately (Sachdeva et al., 2015). Carriers such as MNPs can be encapsulated in liposomes while carrying potential therapeutic molecules such as cDNA, siRNA, and polypeptides, which serves as the drug delivery system (Thomsen et al., 2015).

Of all the drug delivery strategies, SPIONs comprising of maghemite (Fe_2_O_3_) and magnetite (Fe_3_O_4_) prove their advantage in targeted drug delivery systems (Anwar et al., 2014). The characteristics of both CAs and the delivery system make SPIONs the optimal choice for targeting drug delivery systems (Anwar et al., 2014; Dulińska-Litewka et al., 2019).

## The Application of Iron Oxide Nanoparticles in Neurodegenerative Diseases

**Figure 1:**
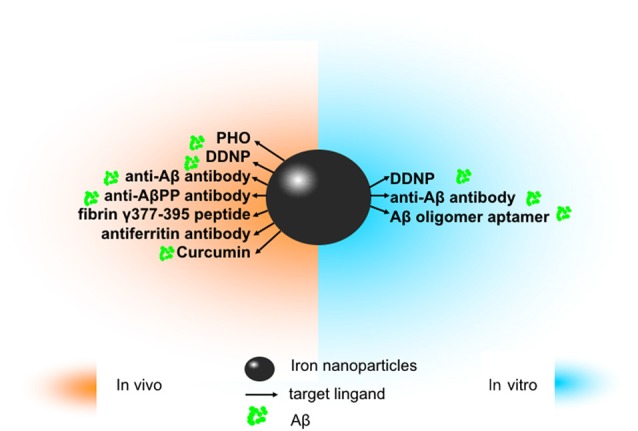
The application of iron oxide nanoparticles (IONPs) in diagnosis and treatment of Alzheimer’s disease (AD). Since amyloidβ (Aβ) has been identified as an ideal imaging biomarker of AD at present, with the help of magnetic resonance imaging (MRI), IONPs can be used for the detection of Aβ to assist in diagnosis and treatment of AD. *In vitro*, magnetic nanoparticles (MNPs) labeled with antibodies against Aβ-40 and Aβ-42 is applicable to detect Aβ in the blood. Conjugated with the Aβ oligomer aptamer and the complementary oligonucleotide of the Aβ oligomer aptamer, IONPs can be developed as a method to measure the Aβ oligomer in the artificial cerebrospinal fluid (CSF). DDNP-superparamagnetic iron oxide nanoparticles (SPIONs) with high affinities to Aβ_(1–40)_ aggregates can be detected by fluorophotometry. *In vivo*, ultrasmall superparamagnetic iron oxide (USPIO)-PHO could mark on amyloid plaques in the NMRI mice brain; anti-Aβ protein precursor (AβPP) antibody-conjugated SPIONs can visualize the number of plaques in AβPP/PS1 transgenic mice. DDNP-SPIONs nanoparticles significantly decrease the signal intensity (SI) in the hippocampal area in the rat AD model. Curcumin-conjugated superparamagnetic iron oxides (SPIOs) can detect amyloid plaques in Tg2576 mice brains. Fibrin γ377–395 peptide-conjugatedγ-Fe_2_O_3_ nanoparticles could specifically inhibit the microglial cells in rTg4510 tau-mutant mice and thus provide a possible therapeutic strategy towards neurodegenerative tauopathies. In addition, magnetic IONPs bound to an antiferritin antibody were developed to detect ferritin protein in areas with a high amount of amyloid plaques in the brain of a transgenic AD mouse model. BBB, blood–brain barrier.

In this section, we simply summarized the application of iron nanoparticles in diagnosis and treatment in neurodegenerative disease, including AD, PD, and ALS. The following parts related to AD are summarized in Figure 1.

### Detecting Iron Deposit in Amyloid Plaques by MRI

The deposition of Aβ is one of the primary histopathological characteristics of AD (Tiwari et al., 2019). With the relatively large size and being located intercellularly, Aβ can be regarded as an ideal imaging biomarker (Zhang et al., 2015). The Aβ deposition is earlier than clinical manifestations and increases gradually with the progress of the disease (Tiwari et al., 2019).

It has been reported that because of the limited brain uptake after the intravenous injection, the styrylbenzenes and the pathological staining dye analogs, such as X-34, ISB, IMSB, Congo Red, and Chrysamine G, are not suitable for the imaging of the Aβ plaque (Zhou et al., 2014). Since there are iron deposits in the amyloid plaques, in T2- and T2*-weighted MR images, the plaques showed a hypointense signal (Chamberlain et al., 2009). Thus, with the help of specific MRI sequences, the detection of amyloid plaques can use not the CAs but the iron content (Ansciaux et al., 2015). Since the detection of iron relies on the nature properties of the amyloid plaques and the stage of the disease (larger plaques contain higher amounts of iron, which makes the detection easier), it normally requires greater than 7-T magnetic field and several hours as acquisition time (Ansciaux et al., 2015). In AD transgenic mice, the levels of iron were high in thalamic plaques and low in cortical/hippocampal plaques, and by using different MRI sequences, the visibility of plaques in the cortex/hippocampus was different from those in the thalamus (Wengenack et al., 2011). The results showed that by T(2)SE, all plaques were detectable equally, but by T(2)*GE pulse sequences, only thalamic plaques were detected reliably (Wengenack et al., 2011). Human AD plaques are similar to cortical/hippocampal plaques of AD mice, and MRI methods that are less dependent on iron magnetic susceptibility effect might be suitable for imaging the human AD plaque (Wengenack et al., 2011). Studies have shown that CAs can conduce to Aβ plaques detecting under the condition of high field MRI both *in vitro* and *in vivo* (Yang et al., 2011a; Petiet et al., 2012).

### Iron Oxide Nanoparticles in the Diagnosis of AD *in vitro*

The effective treatments for AD depend on the detection and quantitation of soluble AD biomarkers as early diagnosis, mainly by measuring the total tau protein and Aβ concentrations in CSF or plasma and detecting a suspected pathogenic biomarker. But being affected by low concentrations and other factors, these two strategies may lead to inconclusive imprecise results (Brambilla et al., 2011).

It has been reported that magnetic reagents that consist of MNPs magnetically labeled with antibodies against Aβ-40 and Aβ-42 can be applied to detect Aβ in the blood (Yang et al., 2011a). By applying the immunomagnetic reduction (IMR) assay, the concentration can be detected, which is lower than 10 pg/ml at Aβ-40 and 20 pg/ml at Aβ-42 and, furthermore, with high specificity (Yang et al., 2011a). Skaat et al. (2013) reported that the fixation of the aAβmAb clone BAM10 to near-infrared fluorescent maghemite nanoparticles enables to detect Aβ_40_ fibrils specifically *ex vivo* by both fluorescence and MRI.

1,1-Dicyano-2-[6-(dimethylamino)naphthalene-2-yl]propene (DDNP) carboxyl derivative-modified SPIONs (DDNP-SPIONs), synthetized by Zhou et al. (2014) have shown high binding affinities toward Aβ_(1–40)_ aggregates by the investigation of fluorophotometry *in vitro* trials. Because of benefits such as versatile surface chemistry, relatively small size, and monodisperse size distribution, DDNP-SPIONs would provide opportunity for the molecular diagnosis of AD (Zhou et al., 2014).

With BaYF5:Yb, Er nanoparticles (UCNPs) as upconversion fluorescence labels, Fe_3_O_4_ MNPs as the recognition and concentration elements conjugated with the Aβ oligomer aptamer and the complementary oligonucleotide of the Aβ oligomer aptamer, respectively (Jiang et al., 2017). The developed method measured Aβ oligomer in artificial CSF successfully (Jiang et al., 2017).

With corresponding antibodies conjugated as targeting ligands, magnetic IONPs coated by antibiofouling polymer polyethylene glycol-block-allyl glycidyl ether (PEG-b-AGE) could capture Aβ_40_ and Aβ_42_ peptides and tau protein in CSF- and serum-mimicking samples, with high specificity (>90%) and sensitivity (>95%; Li et al., 2019). And the antibody-conjugated IONPs also detected Aβs and tau protein from the human whole blood samples, with significantly higher sensitivities than those of antibody-conjugated Dynabeads (Li et al., 2019).

### Iron Oxide Nanoparticles in the Diagnosis of AD *in vivo*

It is reported that in AD transgenic mice, a gadolinium-loaded molecular probe can cross the BBB and specifically bind to the Aβ plaques after intravenous injection, and gained more than ninefold enhancement in the cortex and the hippocampus by using 7-T MRI (Poduslo et al., 2002). But as set forth, ionic gadolinium complexes can leak toxic Gd^3+^ ions with short half-life, and the Gd^3+^ chelates can lead to renal insufficiency.

Another research suggested that after introducing several magnetic CAs in AD transgenic mice to detect amyloid plaques, the USPIONs were able to commendably identify transgenic mice to the wild type by detecting amyloid plaques T2*-weighted in MRI (Yang et al., 2011b). Ansciaux et al. (2015) reported that a USPIO-PHO (USPIO coupled to peptide C-IPLPFYN-C) could cross the BBB of NMRI mice by intravenous injection and accumulates in the brain for 90 min, with high affinity (nanomolar binding affinity) and low toxicity. The half-life of USPIO-PHO was about 3 h. These MRI and histochemistry studies showed that USPIO-PHO had the potential to label amyloid plaques in the brain (Ansciaux et al., 2015). Sillerud et al. (2013) synthesized an anti-AβPP antibody-conjugated SPION, which can cross the BBB and act as an *in vivo* CA for MRI of Aβ plaques in AD. By MRI, the number of plaques visible per brain was about twice in AβPP/PS1 transgenic mice than that in control AD mice (Sillerud et al., 2013). DDNP-SPION nanoparticles were further tested by intrahippocampal injection of Aβ_1–40_ in a rat AD model (Zhang et al., 2015). After intravenous injection of DDNP-SPIONs in AD rats and testing by coronal T2*-weighted images, the signal intensity (SI) detected in the hippocampal area was decreased significantly, which indicated the binding of DDNP-SPIONs to the Aβ plaques (Zhang et al., 2015). Curcumin is a natural compound that can bind to amyloid plaques specifically (Cheng et al., 2015). Cheng et al. (2015) injected curcumin-conjugated SPIOs to Tg2576 mouse and nontransgenic mice; amyloid plaques were detectable in Tg2576 mice brains by *ex vivo* T2*-weighted MRI, but no plaques were found in the control group.

Growing evidence shows that vascular remodeling might be an important factor in the pathophysiologic mechanisms of AD (Klohs et al., 2012). Based on this, Klohs et al. (2012) used contrast-enhanced magnetic resonance microangiography (CE-μMRA) in wild-type control mice and arcAβ mice to estimate the density of the cortical microvasculature before and after the administration of SPIONs. CE-μMRA can be available for visualizing the cerebral arteries and veins whose diameter is less than 60 μm, the nominal pixel resolution (Klohs et al., 2012). The authors take the attitude that the deposition of Aβ and fibrin results in impaired perfusion and vascular occlusion, which may finally contribute to the density reduction of transcortical vessels (Klohs et al., 2012).

In addition to the experiments on animals mentioned above, medical researchers may consider using a new and broader approach, including T1ρ-weighting, macroscopic T2 mapping, SE imaging, FSE imaging, and gradient-echo (GRE) imaging, to detect the plaque in human clinical trials (Chamberlain et al., 2009).

By *ex vivo* MRI (11.7 T), anti-amyloid targeted superparamagnetic IONPs were capable of detecting deposition of amyloid β plaques and neuroinflammation activation by microglia in 3X AD transgenic mice (Tafoya et al., 2017).

Based on magnetic resonance molecular imaging (MRMI), ultrasmall particles of iron oxide (USPIO) functionalized with a disulfide constrained cyclic heptapeptide (PHO) was able to target Aβ plaques and cross the BBB in AD transgenic mice (André et al., 2017). The colocalization of USPIO-PHO with amyloid plaques on brain sections was demonstrated by immunohistochemistry and immunofluorescent experiment (André et al., 2017). Moreover, the amount of amyloid plaques detected by USPIO-PHO was in good correlation with that of plaques detected with anti-amyloid β antibody and Perls’-DAB staining (André et al., 2017).

In a recent study, magnetic IONPs bound to an antiferritin antibody were developed to detect ferritin protein in areas with a high amount of amyloid plaques, in particular the subiculum in the hippocampal area, in the brain of a transgenic mouse model with five familial AD mutations (Fernández et al., 2018). Functionalized IONPs were capable of recognizing and combining specifically to the ferritin protein accumulated in the subiculum area of the AD transgenic mice (Fernández et al., 2018).

### Application of Iron Oxide Nanoparticles in the Treatment of AD

For AD, drug therapy can improve the symptoms but cannot prevent the development of the disease (Busquets et al., 2014). For patients whose disease course beyond an average of 6 months, the benefits of drug therapy are not sustained (Corbett et al., 2012). The drug distribution spreading across the whole brain may decrease the amount of drug reaching the target, thus reducing the effectiveness of the therapeutic. Therefore, a delivery system must be developed to ensure that the drug reaches the exact lesion site. Major efforts have been directed toward developing molecules with high affinity for Aβ, which can reduce the Aβ level in the brain (Brambilla et al., 2011).

It is reported that prominent microglial activation precedes tangle formation, and elimination of tau-induced microglial activation could delay the progression of neurodegenerative tauopathies (Yoshiyama et al., 2007). Adams et al. (2007) conjugated fibrin γ377–395 peptide [one fibrin-derived peptide that can inhibit microglial activity *in vivo* specifically to iron oxide (γ-Fe_2_O_3_) nanoparticles with diameters 21 ± 3.5 nm] in order to counteract the short half-life of the peptide. The study showed that, compared to the free peptide of the same concentration, γ-Fe_2_O_3_ nanoparticles could specifically inhibit the microglial cells in rTg4510 tau-mutant mice, supporting the fact that the nanoparticles can be used for the delivery of substances to the brain and for providing a possible therapeutic strategy to neurodegenerative tauopathies (Glat et al., 2013).

Kouyoumdjian et al. (2013) reported a biomimetic path using glyconanoparticles-SPIO to detect Aβ. The superparamagnetic nature enabled the detection of Aβ both *in vitro* and in mouse brains by MRI. The glyconanoparticles not only can reduce Aβ mediated cytotoxicity damnification to cells greatly but also can highlight the detection and imaging potential of Aβ (Kouyoumdjian et al., 2013).

There are studies describing the effect of magnetic nanoparticulate on Aβ fibrillation. Depending on the size and the surface area, a dual effect on the Aβ fibrillation kinetics was observed, with lower concentrations of SPIONs decreasing the rate of Aβ fibrillation, while higher concentrations enhanced the rate in the aqueous solution (Mahmoudi et al., 2013). Consistent with the previous study, Mirsadeghi et al. (2016) showed that lower concentrations of SPIONs coated by PEG-NH2 inhibited the process of Aβ fibrillation under magnetic field, whereas high concentrations accelerated the process. Furthermore, the coating charge also exerts a considerable effect on the Aβ fibrillation process. In comparison with the negatively charged or uncharged SPIONs, lower concentrations of SPIONs with positive coating charge promoted the fibrillation (Mirsadeghi et al., 2016). By applying thioflavin-T fluorescence emission, the effect of SPIONs with different electric charges on both β-amyloid and α-synuclein fibrillation process was investigated. The negatively charged nanoparticles encoded to -COOH by dextran-coating decreased the binding level of thioflavin-T particles to β-sheets (Javdani et al., 2019).

Combining nerve growth factor (NGF) and quercetin with superparamagnetic IONPs promoted neurite outgrowth and increased the complexity of the neuronal branching trees in PC12 cells, as potential therapeutics for neurodegenerative diseases (Katebi et al., 2019).

Sonawane et al. (2019) screened out one kind of protein-capped (PC) metal nanoparticles that inhibit Tau aggregation *in vitro* for the first time. They proved that because of the increased reactive oxygen species production and the resulting oxidative stress, the uncapped CdS nanoparticles make themselves toxic to HeLa cells and bacteria, but by capping, these CdS can obtain an entirely different property and become more biocompatible; thus, the biosynthetic PC metal nanoparticles, particularly iron oxide, will not influence the viability of neuroblastoma cells (Sonawane et al., 2019).

### Application of Iron Oxide Nanoparticles in the Diagnosis of PD

Yang et al. (2016) invented a reagent for IMR consisting of antibodies against α-synuclein functionalized with MNPs. By using an ultrasensitive immunoassay utilizing IMR, the α-synuclein detection dynamic range is from 0.3 fg/ml to 310 pg/ml in plasma (Yang et al., 2016). The nanoparticles can differentiate PD patients, PDD (PD dementia) patients, and healthy subjects depending on the significantly different concentration of plasma α-synuclein (Yang et al., 2016).

Studies have conjugated multimodal IONPs to Rhodamine-B (MION-Rh), and then labeled with MSCs from umbilical cord blood (UC-MSC; Sibov et al., 2014). Labeled cells were infused into the striatum of PD adult male rats, and 15 days later by T2 MRI, the cells were observed migrating along the medial forebrain bundle to the substantia nigra as hypointense spots (Sibov et al., 2014).

### Application of Iron Oxide Nanoparticles in the Treatment of PD

Gene therapy, which targets the expression of α-synuclein in neurons, is of great concern, and shRNA (short hairpin RNA) has been identified as a promising treatment of PD (Niu et al., 2017). Therefore, researchers coated magnetic Fe_3_O_4_ nanoparticles with oleic acid molecules as a nanocarrier and absorbed shRNA. They demonstrated that these superparamagnetic nanoparticles can reduce the expression of α-synuclein and thus can prevent the toxic effects on the cell and suppress apoptosis by α-synuclein (Niu et al., 2017).

Salama et al. (2017) isolated MSCs from C57BL/6 mice, incubated MSCs with micrometer-sized iron oxide (referred to as MPIOs) particles, and finally administrated them in a PD mouse model by the way of the intranasal (IN) route. In the experiment, MPIO-labeled MSCs were used as stem cell tracking stained with Prussian blue (Salama et al., 2017). The following histopathological evaluation by positive Prussian blue staining revealed the successful delivery of MSCs (Salama et al., 2017). The neurobehavioral assessment was improved following MSC administration (Salama et al., 2017).

Studies have confirmed that after receiving human ventral mesencephalic NSC (hVM1) grafts, parkinsonian animals showed an amelioration in resting tremor and cognitive performance (Kouyoumdjian et al., 2013) Based on this, Ramos-Gómez et al. (2015) believed that hVM1 cells and their derivatives represented a helpful method for cell therapies focused on neurodegenerative diseases, PD in particular. In the study, they found that MNPs of different sizes (with a diameter of 50 and 100 nm) were labeled with hVM cells nearly 100% in defect of any transfection agents (Ramos-Gómez et al., 2015). After transplanting MNP-labeled hVM cells into the striatum of the PD rat model, MNPs were distributed evenly throughout the transplant region detected by histological analysis (Ramos-Gómez et al., 2015). The researchers used MNPs to label hNSCs (human NSCs) and injected them into hemiparkinsonian rats in order to follow stem cell fate over time to verify the efficient application of stem cells after transplantation (Ramos-Gómez et al., 2015). The result shows that by the use of MRI, the MNP-labeled hNSCs grafted into hemiparkinsonian rats can be successfully visualized up to 5 months after transplantation at different time points (Ramos-Gómez et al., 2015).

A recent study has demonstrated that dextran-coated IONPs (Dex-IO NPs) can improve the therapeutic effects of human MSCs in a mouse model of PD (Chung et al., 2018). The loss of dopaminergic neurons was decreased and the migration capacity and the differentiation of human MSCs to dopaminergic neurons were enhanced (Chung et al., 2018). Therefore, Dex-IO NPs can be considered as a promising carrier for MSC therapy for PD.

### Application of Iron Oxide Nanoparticles in the Diagnosis of ALS

Evans et al. (2014) suggested that T2-weighted MRI offered a strong biomarker potential in superoxide dismutase (SOD1) G93A transgenic ALS mouse model. They put VCAM-1 (vascular cell adhesion molecule 1, one cellular adhesion molecule that can upregulate during endothelial activation) together with MPIO (microparticles of iron oxide; Evans et al., 2014). However, they concluded that VCAM-MPIO as a biomarker in *SOD1* ALS is useless (Evans et al., 2014).

### Application of Iron Oxide Nanoparticles in the Treatment of ALS

The therapeutic efficacy of SkmSCs (subpopulation of human skeletal muscle–derived stem cells) with MSC-like features was evaluated after intracerebroventricular injection to the Wr mouse (Wobbler mouse, the most typical model of spontaneous motor neuron degeneration) by Canzi et al. (2012). The research team confirmed that MRI can visualize stem cells and follow their migration after transplantation in the CNS of rodents (Canzi et al., 2012). Bigini et al. (2012) traced amniotic fluid cells (hAFCs) in a chronic neurodegenerative/inflammatory environment in a similar approach; after SPION loaded hAFC administration, the signal in the ventricles of the brain became hypointense in both healthy and Wr mice. All the abovementioned indicate that SPION can be a tracer to monitor the efficacy of stem cell therapy for ALS.

## Summary

For neurodegenerative diseases such as AD, PD, and ALS, the early diagnosis toward these diseases is difficult. In order to improve the symptoms by drug therapy based on the diagnosis at the early stage of the disease, MRI CAs, especially SPIO and USPIO, have already been developed in imaging and targeting drug delivery. Despite the fact that regulatory bodies have already approved some SPIO agents for many years, the clinical application of these agents has been proven a difficult journey (Wáng and Idée, 2017). For instance, by the end of 2015, only five types of SPIO had been designed and applied in clinical settings as magnetic resonance CAs (Yang et al., 2016). Of these, one is available only in a few countries; the other four have already been terminated in the follow-up study or even pulled out of the market (Yang et al., 2016). The mandatory requirements of nanoparticles should be improved, including biocompatibility, biodegradability, biodistribution, stability under physiological conditions, accurate pharmacokinetics, and minimal side effects. Designing nanoparticles to pass through the BBB is even more challenging and complex than conventional drug delivery. Major efforts should be made to improve targeted drug release and therapeutic efficacy, and to increase sensitivity and specificity of noninvasive imaging. And the olfactory pathway provides a noninvasive route for nanoparticles to enter the CNS. Even if the noninvasive imaging examination method cannot be applied to the human body temporarily, they can be used to the mouse model to explore new diagnostic and therapeutic methods for neurodegenerative diseases. It is not too far off before IONPs hold promise for the development of early diagnosis and the accomplishment of the aim of personalized therapy toward neurodegenerative diseases.

## Author Contributions

SL, CM, M-QZ, and W-NJ searched the literature and drafted the manuscript. XW and YY critically revised the manuscript. All authors listed have made a substantial, direct, and intellectual contribution to the work, and approved it for publication.

## Conflict of Interest

The authors declare that the research was conducted in the absence of any commercial or financial relationships that could be construed as a potential conflict of interest.

## Abbreviations

Aβ: amyloid β
AD: Alzheimer’s disease
ALS: amyotrophic lateral sclerosis
BBB: blood–brain barrier
CA: contrast agent
CNS: central nervous system
CSF: cerebrospinal fluid
IONP: iron oxide nanoparticle
MNP: magnetic nanoparticle
MRI: magnetic resonance imaging
PET: positron emission tomography
PD: Parkinson’s disease
USPIO: ultrasmall superparamagnetic iron oxide
SPIO: superparamagnetic iron oxide
SPION: superparamagnetic iron oxide nanoparticle.
